# Intramuscular Fat in the Medial Gastrocnemius Muscle of People Who Have Had a Stroke

**DOI:** 10.3389/fbioe.2020.00613

**Published:** 2020-06-09

**Authors:** Arkiev D'Souza, Bart Bolsterlee, Robert D. Herbert

**Affiliations:** ^1^NeuRA, Randwick, NSW, Australia; ^2^School of Medical Sciences, University of New South Wales, Randwick, NSW, Australia; ^3^Graduate School of Biomedical Engineering, University of New South Wales, Randwick, NSW, Australia

**Keywords:** intramuscular fat, stroke, contracture, mDixon MRI, medial gastrocnemius

## Abstract

**Objective:** To compare intramuscular fat fraction in people who have ankle contractures following stroke with the intramuscular fat fraction in control participants.

**Design:** mDixon MRI images were used to quantify intramuscular fat fractions in the medial gastrocnemius muscles of people who had experienced a hemiparetic stroke (*n* = 14, mean age 60 ± 13 years) and control participants (*n* = 18, mean age 66 ± 12 years).

**Results:** Intramuscular fat fractions were similar in the paretic and non-paretic sides of stroke patients (mean on paretic side 14.5%, non-paretic side 12.8%, difference 1.6%, 95% confidence interval −0.7 to 4.1%). The intramuscular fat fraction on the paretic side was higher than in the control group (mean intramuscular fat fraction in control muscles 7.6%; difference 7.8%, 95% confidence interval 4.6–10.9%). The difference between intramuscular fat fractions in non-paretic and control legs increased with age. Body mass index was similar in stroke patients and controls. There was no association between medial gastrocnemius intramuscular fat fraction and dorsiflexion range.

**Conclusion:** Muscles of stroke patients had elevated intramuscular fat fractions compared to muscles from control participants which were not explained by differences in body mass index. There is no clear relationship between intramuscular fat in the medial gastrocnemius muscle and dorsiflexion range of motion.

## Introduction

Contracture is a loss in joint range of motion caused by the increase in passive stiffness of muscles (Fergusson et al., 2006; Harvey et al., 2017). Contractures can severely compromise the ability to execute activities of daily living (O'Dwyer et al., 1996). People who have had a stroke often develop ankle joint contractures (Kwah et al., 2012a). It is difficult to treat contractures because the mechanisms that lead to the increased muscle stiffness are poorly understood.

In addition to contracture, stroke patients also commonly experience muscle weakness. Muscle weakness can be due to central mechanisms such as impaired descending drive to the muscle, but it may also be due to alterations at the periphery, such as reduced muscle size and physiological cross-sectional area. Alterations within the muscle, such as the infiltration of fat, reduce the cross-sectional area of muscle fibers per unit cross-sectional area of muscle, compounding to the deleterious effects of reduced muscle volume on muscle strength. Furthermore, computer simulations suggest that the presence of intramuscular fat increases muscle stiffness (Rahemi et al., 2015), implying that the proliferation of intramuscular fat could contribute to the development of both contractures and muscle weakness.

Several studies have investigated fat content in limbs of stroke patients. People who have had a stroke tend to have more intramuscular fat than people who have not had a stroke (Akazawa et al., 2018). It has been reported that the paretic side has more fat (Iversen et al., 1989; Jorgensen and Jacobsen, 2001), or a greater proportion of intramuscular fat (Ryan et al., 2002) than the non-paretic side, although one study found more intramuscular fat in non-paretic than paretic muscles (Ryan et al., 2011). Measurements of fat have been made using dual-energy X-ray absorptiometry (DEXA) (Iversen et al., 1989; Jorgensen and Jacobsen, 2001), ultrasound (Akazawa et al., 2018), computed tomography (CT) (Ryan et al., 2002, 2011), and T1-weighted magnetic resonance imaging (MRI) (Ramsay et al., 2011). All these techniques have limitations for quantifying intramuscular fat content. While DEXA can be used to find limb-specific fat content, it cannot be used to measure muscle-specific fat content. The validity of ultrasound fat quantification has not been demonstrated. T1-weighted MRI and CT do not directly measure fat but instead classify voxels as fat based on an image intensity threshold taken from voxels which are known to be comprised of fat, such as subcutaneous tissue. This can be problematic when in-field inhomogeneities are present (Burakiewicz et al., 2017) (i.e., when there is variation in signal intensity which is not due to fat content). While CT measurements are capable of obtaining measurements from a whole muscle, studies that have investigated intramuscular fat content in people who have had a stroke have obtained measurements from a single cross-sectional slice (Ryan et al., 2002) or a few slices (Ryan et al., 2011) which may not be representative of the whole muscle.

An alternative MRI technique called mDixon imaging can also be used to measure intramuscular fat (Kovanlikaya et al., 2005b; Wren et al., 2008; Karampinos et al., 2012; Noble et al., 2014a; Burakiewicz et al., 2017). This imaging technique exploits the difference in the resonant frequencies of protons in water and lipids to measure fat fractions in each voxel. mDixon scans can accurately (Kovanlikaya et al., 2005a; Fischer et al., 2014; Noble et al., 2014b) and reliably (Ponrartana et al., 2014) quantify muscle-specific, whole-muscle fat fractions *in vivo*. This technique has already been used to quantify intramuscular fat in populations with CP (Noble et al., 2014a) and muscular dystrophy (Burakiewicz et al., 2017), but has not yet been used to investigate intramuscular fat in people who have had a stroke.

In this study, mDixon imaging was used to compare the intramuscular fat fractions in the medial gastrocnemius muscles from the paretic and non-paretic sides of people with ankle contracture following hemiparetic stroke. Comparisons were also made between people who have and have not had a stroke. The medial gastrocnemius muscle was chosen in this study because stiffness of this muscle contributes to ankle joint contractures after stroke (Kwah et al., 2012b), and because of its functional significance in locomotion.

## Materials and Methods

We further analyzed MRI data obtained from the legs of 14 hemiparetic stroke patients and 18 control participants. We have previously reported analyses of muscle architecture in these participants (D'Souza et al., 2020) but have not previously published analyses of intramuscular fat.

All procedures were approved by the local human research ethics committee (HC number: HC15006). Written informed consent was obtained from all participants before participation.

### Participants

Participants included in this study were at least 18 years of age and had loss of ankle range of motion resulting from stroke (stroke group), or had not had a stroke (control group). Where possible, an initial assessment was conducted to identify patients with contracture, defined as having <5° passive dorsiflexion with the knee extended as measured with a goniometer by an experienced physiotherapist. In most cases it was not possible to conduct the initial assessment because participants were not referred to the study by a physiotherapist. Such participants were screened over the telephone, and were eligible for participation if they self-identified (or were identified by a carer) as having a stiff ankle as a result of stroke. Additional inclusion criteria include having no recent history of orthopedic surgery or musculoskeletal injury, and an exclusion criterion was having received a botulinum toxin injection in the 6 months prior to the experiment. Participants were not excluded based on the severity of stroke, time since stroke, or walking ability.

### Range of Motion Measurement

**Figure 1:**
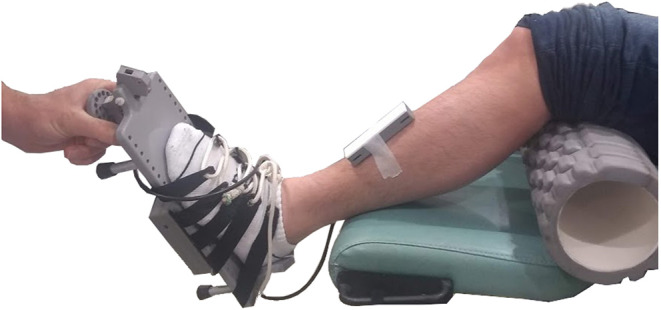
The custom-built electronic goniometer used to measure dorsiflexion range. The participant was placed in a supine position. An 11 cm diameter foam roller was placed under the knee. The experimenter rotated the foot through dorsiflexion until 10 Nm was reached. Dorsiflexion range was defined as the angle between an accelerometer placed on the tibia and an accelerometer placed underneath the sole of the foot at 10 Nm of torque. Ankle angles below 90° indicate plantarflexion and above 90° dorsiflexion.

A custom-built electronic goniometer was used to measure passive dorsiflexion range (Figure 1). Dorsiflexion range was defined as the ankle angle between an accelerometer on the tibia and an accelerometer underneath the sole of the foot with 10 Nm of torque applied to the sole of the foot. Ankle angles below 90° indicate plantarflexion and above 90° dorsiflexion.

The foot was strapped in a footplate equipped with a force transducer. The experimenter applied increasing force to the sole of the foot. While speed of rotation was not controlled, the same experimenter conducted the measurement on all participants. The measurement was taken with the participant lying supine and with a foam roller (11 cm diameter) positioned under the knee. Ten preconditioning rotations were conducted to minimize history-dependent effects, followed by five recordings. The mean of the five measurements was used as the participant's dorsiflexion range.

### MRI Image Acquisition

Participants were positioned supine on the MRI scanner bed. A foam wedge was placed under the knee to prevent deformation of the calf from the weight of the shank. The participant was asked to remain relaxed during the scan. The left and right lower legs were scanned in separate acquisitions within the same scanning session.

A 3-Tesla MRI scanner (Achieva TX; Philips Medical Systems, Best, The Netherlands) with a 32-channel cardiac coil was used to obtain mDixon MRI images of both lower legs. A 3D multi-echo mDixon Fast Field Echo (FFE) scan was used. Three-hundred transverse anatomical slices were obtained covering the entire cross-section of the shank from the distal end of the femur to the ankle. The settings were: 2-point 3D mDixon FFE, TR/TE1/TE2 6.1/3.5/4.6 ms, field of view 180 × 180 mm, slice thickness 2 mm (1 mm over contiguous), acquisition matrix 180 × 180 (reconstructed to 192 × 192), reconstructed voxel size 0.94 × 0.94 × 1 mm, number of signal averages (NSA) 2 and scan time 164 s.

### Intramuscular Fat Calculation

**Figure 2:**
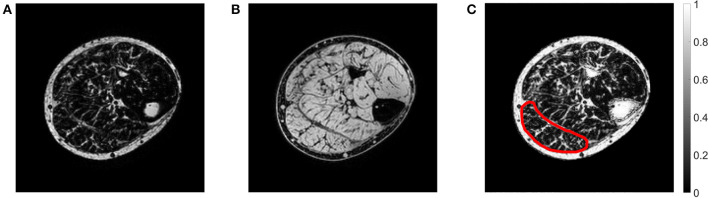
Example of an axial mDixon image from the paretic leg of a stroke patient showing the **(A)** fat-saturated image, **(B)** water-saturated image, and **(C)** fat fraction map. In the fat fraction map, voxels with high fat fractions are white and voxels with low fat fractions are black. The slice is approximately midway between the ankle and the knee. The medial gastrocnemius muscle boundary has been outlined.

The mDixon imaging technique exploits the chemical shift difference between water and fat to produce an in-phase and out-of-phase image, from which a separate fat-saturated (Figure 2A) and water-saturated (Figure 2B) image can be obtained (Dixon, 1984). A fat fraction map (Figure 2C) was calculated using the ratio of water and fat signal intensities within each voxel:

$$\begin{array}{l} fat\;fraction=\;\frac{I_{fat}}{I_{fat}+I_{water}}\;\times 100\%\; \end{array}$$

where I_fat_ is the signal intensity from the fat-saturated image and I_water_ is the signal intensity from the water-saturated image.

The medial gastrocnemius muscle was manually outlined on all of the slices of the mDixon scan (on the out-of-phase image) using image processing software ITK-SNAP (Yushkevich et al., 2006). Segmentations were performed by a researcher with experience in segmenting muscles of the lower leg.

The intramuscular fat fraction of the medial gastrocnemius was calculated as the average fat fraction of all voxels in the muscle. Voxels at the boundary of the muscle were excluded from calculating the average to remove potential inaccuracies from partial volume effects and small segmentation errors. For participants who have had a stroke, separate measurements were obtained for the paretic and non-paretic side. For control participants, fat fractions of the left and right muscle were averaged.

### Statistical Analysis

A sample size of 32 subjects is sufficient to detect a between-group difference in intramuscular fat fractions of 1 SD with a probability of ~80%.

In the primary analysis, linear mixed models were used to compare the differences in mean intramuscular fat fraction between the paretic, non-paretic and control muscles. Participants were assigned random intercepts and age and group were modeled as fixed effects. We also determined whether the rate of change of intramuscular fat fraction with respect to age was the same across groups by adding an age by group interaction to the model.

Linear mixed models were also used to compare the mean differences between groups for dorsiflexion range of motion. Participants were assigned random intercepts and group was modeled as a fixed effect.

Secondary analyses explored the linear correlation between intramuscular fat fraction of the medial gastrocnemius and body mass index (BMI) and ankle dorsiflexion range. Separate correlation analyses were conducted for each group.

An additional linear mixed model was used to explore if the differences in mean intramuscular fat fraction between the paretic and non-paretic muscles varied with time since stroke. Participants were assigned random intercepts, and instead of age, time since stroke was modeled as a fixed effect along with group. We also determined whether the rate of change of intramuscular fat fraction with respect to time since stroke was the same across groups by adding a time since stroke by group interaction term to the model.

All statistical analyses were conducted using Matlab (version R2017b, The MathWorks Inc., Natick, Massachusetts, United States) statistics toolbox.

## Results

### Participant Characteristics

Participant characteristics are shown in Table 1. No significant differences were detected between groups for age, height, weight, or BMI (unpaired *t*-test, *P* > 0.05 for all variables).

**Table 1:** Participant characteristics.

	**Stroke (*n* = 14)**	**Control (*n* = 18)**	***P***
Age (years)	60 (13)	66 (12)	0.15
Gender (male/female)	10/4	15/3	0.42
Height (cm)	173 (7)	174 (9)	0.91
Weight (kg)	85 (18)	79 (14)	0.29
BMI (kg/m^2^)	26 (7)	28 (4)	0.22
Time since stroke (years)	5 (4)	-	-
Paretic side (left/right)	7/7	-	-

*Values are means (SD). An unpaired t-test was used to compare groups. A chi-square test was used to compare group differences for gender*.

On average, the paretic, non-paretic, and control groups had 97° (*SD* 9°), 108° (8°), and 104° (7°) range of motion, respectively. On average, the paretic side of stroke patients had 11° (95% confidence interval 8–13°) less dorsiflexion range than the non-paretic side, and 7° (1–13°) less dorsiflexion range than in control participants. There was no significant difference in range of motion between the non-paretic and control legs (difference −4.0°, 95% confidence interval −9.1 to 1.2°).

### Whole-Muscle Intramuscular Fat

The mean (*SD*) intramuscular fat fraction on the paretic and non-paretic sides were 14.5% (6.3%) and 12.8% (7.4%), respectively. The mean intramuscular fat fraction of the control medial gastrocnemius muscles was 7.6% (2.6%).

**Figure 3:**
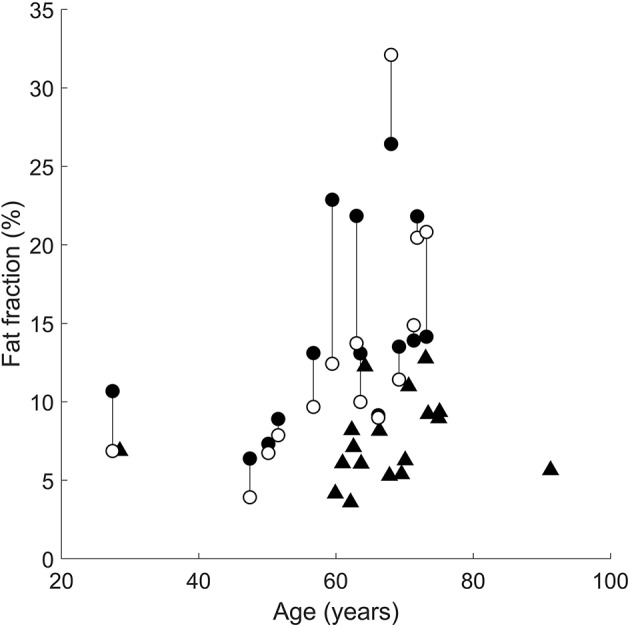
Intramuscular fat fractions in the paretic (filled circles), non-paretic (open circles), and control (triangles) medial gastrocnemius muscles. Vertical lines join paretic and non-paretic muscles from individual stroke patients.

The following comparisons report the *absolute* difference in intramuscular fat fraction (in % of total muscle volume) of the medial gastrocnemius muscle between groups. In all but three patients, the paretic medial gastrocnemius muscle had a higher fraction of intramuscular fat than the non-paretic muscle (Figure 3). However, the difference in intramuscular fat fraction between the paretic and non-paretic muscles was not significant (difference 1.6%, 95% confidence interval −0.7 to 4.1%, *P* = 0.2). On average, the proportion of intramuscular fat in the paretic medial gastrocnemius muscles was 7.8% (4.6–10.9%, *P* < 0.001) higher than the control muscles (Figure 3). In the non-paretic versus control comparison the proportion of intramuscular fat in the non-paretic muscles was 6.5% (3.0–9.9%, *P* < 0.001) higher than the control muscles. However, in this comparison, the age by group interaction term was significant (*P* = 0.01), i.e., the difference between groups depended on age. At age 63 (the average age of all participants), the proportion of intramuscular fat in the non-paretic leg was 6.5% higher than the control leg.

In the comparison between the paretic and non-paretic groups, the interaction term was not significant, meaning that the rate of change of fat per year was the same for both groups. That is, both the paretic and non-paretic groups accumulated intramuscular fat at 0.3%/year (0.1–0.5%/year, *P* = 0.01).

**Figure 4:**
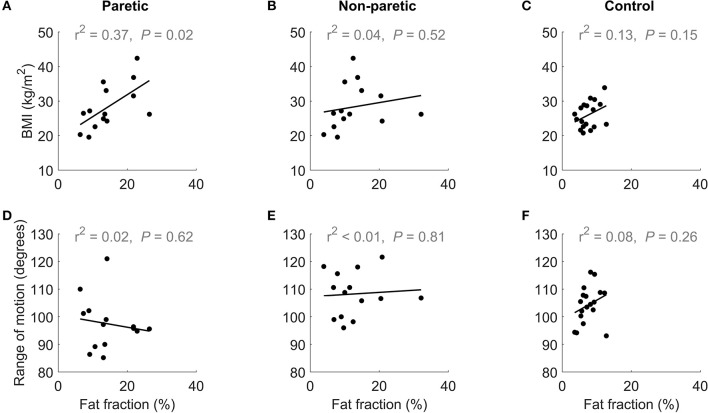
The upper row shows the relationship between BMI and medial gastrocnemius fat fraction in the **(A)** paretic and **(B)** non-paretic muscles of stroke patients, and **(C)** control participants. The lower row shows the relationship between of dorsiflexion range of motion and medial gastrocnemius fat fraction in the **(D)** paretic and **(E)** non-paretic muscles stroke patients, and **(F)** control participants.

Increases in intramuscular fat fraction were positively correlated with BMI in the paretic leg (*r*^2^ = 0.37, *P* = 0.02), but not in the non-paretic and control leg (Figures 4A–C and Table 2). Range of motion did not correlate with intramuscular fat fraction in the paretic, non-paretic or control groups (Figures 4D–F and Table 2).

**Table 2:** Regression coefficient [95% confidence interval] of BMI and range of motion on intramuscular fat fraction in the paretic, non-paretic, and control medial gastrocnemius muscles.

	**Paretic**	**Non-paretic**	**Control**
BMI	0.6 [0.1, 1.2]	0.2 [−0.4, 0.7]	0.5 [−0.2,1.2]
Range of motion	−0.2 [−1.2, 0.7]	0.08 [−0.6, 0.8]	0.7 [−0.6, 2.0]

**Figure 5:**
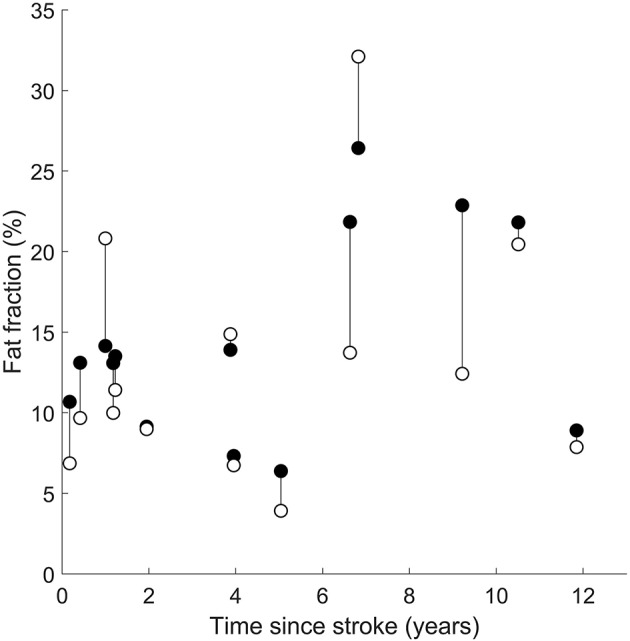
Intramuscular fat fractions in the paretic (filled circles) and non-paretic (open circles) medial gastrocnemius muscles after a stroke. Vertical lines join paretic and non-paretic muscles from individual stroke patients.

There was no effect of time since stroke on the difference in intramuscular fat fraction between the paretic and non-paretic muscles. The interaction between time since stroke and group was not significant (Figure 5).

## Discussion

The proportion of intramuscular fat in people who have had a stroke was compared to control participants using mDixon imaging. While the stroke patients had a moderate level contracture on the paretic side (Andersson and Cocchiarella, 2000), there was no difference in intramuscular fat fraction between the paretic and the non-paretic muscles of stroke patients. Muscles from both the paretic and non-paretic side had a higher proportion of intramuscular fat than the muscles from control participants. There was no difference in intramuscular fat fraction between the paretic and the non-paretic muscles of stroke patients.

Both the paretic and non-paretic muscles in the stroke group had a higher proportion of intramuscular fat than the control group. Reports of adaptations in both paretic and the non-paretic muscles after stroke are not new; Hunnicutt and Gregory showed that both sides have deficits in muscle size and strength compared to people who have not had a stroke (Hunnicutt and Gregory, 2017). It is not known why both sides undergo adaptations. One possible explanation is that decreased neural drive to the paretic side causes physical disability, and consequently, the non-paretic leg undergoes adaptation in response to disuse. This would imply that adaptations to the non-paretic side are not necessarily a direct consequence of having had a stroke, rather, it is an effect of being less mobile after stroke.

People who have had a stroke often have strength deficits. At the periphery, the physiological cross-sectional area (PCSA) of muscle will largely determine strength. The presence of intramuscular fat is likely to exacerbate muscle weakness caused by reduced muscle PCSA by reducing the proportion of the muscle cross-section composed of contractile proteins. The potential detrimental impact of intramuscular fat on muscle quality has previously been simulated (Rahemi et al., 2015). Strength was not measured here, but future studies may consider combining intramuscular fat measurements with strength tests and electromyography to identify whether muscles with higher fat fractions are less able to generate force.

Obese people tend to have more fat in their muscles (Malenfant et al., 2001) so it is possible that the increased amounts of intramuscular fat in the stroke group is due to obesity rather than stroke. However, the stroke group and control groups had a similar mean BMI (26 and 28 kg/m^2^, respectively; *P* = 0.68; unpaired *t*-test) suggesting that the elevated intramuscular fat fractions in the stroke group are not due to obesity. In the correlation analysis of BMI with intramuscular fat, intramuscular fat fraction in the paretic muscle increased with BMI (Figure 4A). However, in the non-paretic muscle, BMI did not increase with fat fraction (Figure 4B), suggesting that factors other than obesity led to the increased intramuscular fat fractions in the stroke group.

In healthy populations, aging is accompanied by intramuscular fat infiltration (Marcus et al., 2010). There was no difference in the mean ages of the stroke and control group, so it is unlikely that age confounded these findings. The slopes of the linear mixed models provide an estimate for the rate of change of intramuscular fat fraction with respect to age, although a longitudinal study would be more appropriate to investigate age related changes in intramuscular fat. In all three comparisons, intramuscular fat fraction increased with respect to age, albeit slowly. These findings support the notion that intramuscular fat increases with age, but should be interpreted with caution given the cross-sectional design.

Time since stroke did not change the mean difference in intramuscular fat fraction or the rate of increase in intramuscular fat fraction between paretic and non-paretic muscles (Figure 5). This finding suggests that intramuscular fat infiltration on both legs is driven by similar stimuli after stroke. Intramuscular fat infiltration did not increase with respect to time since stroke, implying that the intramuscular fat infiltration occurs either rapidly around the time of stroke, or that intramuscular fat fraction was elevated prior to the stroke and did not change with time.

Muscle contracture is thought to be caused by an increase in muscle stiffness (Gao et al., 2009; Kwah et al., 2012b). Intramuscular fat could contribute to contracture by increasing muscle stiffness (Rahemi et al., 2015). While muscle stiffness was not measured directly, range of motion was measured as a surrogate measure of muscle stiffness. Medial gastrocnemius intramuscular fat fraction did not correlate with dorsiflexion range of motion (Figures 4D–F).

Intramuscular fat fractions should not be mistaken with the volume of fat within the muscle. Intramuscular fat fractions are a proportion (often reported as a percentage), while the fat volume is the absolute amount of fat (reported in cm^3^ or mL). We investigated whether the fat volume was different between the three groups by multiplying muscle volume by intramuscular fat fraction. Fat volume in the paretic, non-paretic and control groups were 28.9 cm^3^ (*SD* 14.9 cm^3^), 29.4 cm^3^ (19.9 cm^3^), and 18.2 cm^3^ (9.6 cm^3^), respectively. Fat volume comparisons followed similar trends to the intramuscular fat fraction comparisons: medial gastrocnemius fat volume did not differ between the paretic and non-paretic muscles (−0.5 cm^3^, −6.3 to 5.4 cm^3^). The paretic muscles had 13.0 cm^3^ (4.7–21.4 cm^3^) more fat than the control muscles, and the non-paretic muscles had 13.5 cm^3^ (3.0–24.0 cm^3^) more fat than the control muscles. Thus, people who have had a stroke have both higher proportions and total volume of fat in their medial gastrocnemius compared to people who have not had a stroke. That is despite the muscles of stroke patients having, on average, 15% smaller muscle volumes (D'Souza et al., 2020).

This study demonstrates that intramuscular fat fraction and the absolute volume of fat is elevated in people who have had a stroke. While this finding is not new, the imaging modality used here can accurately and reliably quantify muscle-specific intramuscular fat across the entire muscle. Previous studies used ultrasound [which has only been minimally validated for measurements of intramuscular fat (Young et al., 2015)], DEXA scans (which cannot be used to measure muscle-specific fat fraction), and CT and T1-weighted images (which only indirectly estimate intramuscular fat fraction via reference to fatty voxels).

The measurements presented here along with the previous intramuscular fat measurements paint a consistent picture of elevated intramuscular fat in people who have had a stroke. However, more research needs be done to understand if stroke *causes* the increase in intramuscular fat, or whether the intramuscular fat was already present prior to stroke. It is possible that the stroke cohort had elevated levels of intramuscular fat which preceded, and therefore was not caused by, having had a stroke. If elevated intramuscular fat is detrimental to longevity and quality of life, therapy should be targeted toward reducing intramuscular fat. In people who have had a stroke, this may be achieved by resistance training which reduces intramuscular fat in the paretic and non-paretic lower extremities (Ryan et al., 2011).

### Study Limitations

The range of motion test combines the stiffness of all structures at the ankle and does not provide muscle specific stiffness. As such, the lack of correlation between range of motion and intramuscular fat fraction should not imply that intramuscular fat fraction and muscle stiffness are unrelated. This limitation may be overcome by combining imaging modalities such as shear wave elastography with mDixon imaging to directly measure muscle-specific stiffness and quantify intramuscular fat.

Another limitation of this study is that physical activity was not measured. Physical *inactivity* has been shown to be a risk factor for stroke (O'Donnell et al., 2010) while physical *activity* can prevent infiltration of intramuscular fat in older adults (Goodpaster et al., 2008). The participants who have had a stroke may have had elevated intramuscular fat from being less physically active (which also put them at greater risk of having a stroke). With this cross-sectional dataset, it is impossible to determine whether the elevated intramuscular fat is due to the stroke, or physical inactivity, or a combination of both. Future intramuscular fat studies should consider collecting data on physical activity prior to and after stroke, in addition to physical activity of control participants.

The time since stroke analysis revealed that adaptations do not worsen with time after stroke. This finding was inferred from a relatively small cross-sectional sample—a large longitudinal study would provide a better assessment of changes in intramuscular fat. However, if the finding of no change in intramuscular fat fractions after stroke is true, it would mean that the adaptations were present at the time of stroke and may have been present prior to the stroke. As previously highlighted, physical activity levels were not recorded, although they are likely to be related; differences in physical activity between the stroke cohort (before or after stroke) and control participants could implicate physical activity as a confounder to the elevated intramuscular fat findings. Conversely, a finding of no difference in physical activity between the stroke cohort (prior to stroke) and control participants would suggest that the elevated intramuscular fat levels in people who had a stroke were not due to physical activity levels.

## Data Availability Statement

The raw data supporting the conclusions of this article will be made available by the authors, without undue reservation, to any qualified researcher.

## Ethics Statement

The studies involving human participants were reviewed and approved by the University of New South Wales human research ethics committee (HC15006). The patients/participants provided their written informed consent to participate in this study.

## Author's Note

The manuscript is a chapter in AD thesis. We have previously reported analyses of muscle architecture in these participants (D'Souza et al., 2020). but have not previously published analyses of intramuscular fat. Intramuscular fat measurements were presented in a poster format at the First International Motor Impairment Conference, Sydney, November 2018.

## Author Contributions

RH, BB, and AD conceived and designed the research. AD and BB performed the experiments. AD processed the data and prepared the first draft of the manuscript, which was read and commented on by all authors.

## Conflict of Interest

The authors declare that the research was conducted in the absence of any commercial or financial relationships that could be construed as a potential conflict of interest.
